# Understanding Functional Mobility and Quality of Life Among Isolated Aging Population in the US

**DOI:** 10.1093/geroni/igab046.3445

**Published:** 2021-12-17

**Authors:** Jessika Dayrit, Ana maria Pasatiempo, Kevin Hitosis

**Affiliations:** 1 Washington University in St. Louis, Tamuning, Guam, United States; 2 Department of Veterans Affairs, Department of Veterans Affairs, Alaska, United States; 3 Department of Veterans Affairs, Agana Heights, Guam, United States

## Abstract

**Background:**

Social isolation among older adults linked to serious health conditions. However, little is known if functional mobility among isolated aging adults impacts quality of life. Objectives: This study will examine the association between levels of functional mobilities and risks for developing depression, poor health status, and physical inactivity by controlling socioeconomic factors.

**Methods:**

This is a cross-sectional study, using Behavioral Risk Factor Surveillance System (BRFSS) data 2017. Target population are over age 65 who are living alone (N=50,784). Outcome variables are depression, self-reported health status, and physical inactivity whereas main predictors are activities of daily living (ADL) and instrumental activities of daily living (IADL) controlling for gender, race, marital status, employment status, annual income, and educational level. Weight adjustment analysis and logistic regression were conducted.

**Results:**

Depression, self-reported poor health status, and physical inactivity are higher among isolated aging adults who have limitations with ADL/IADL. Specifically, those with difficulties concentrating/decision making (OR=3.62; CI=3.35-3.91) have higher chance of developing depression than those who do not have this limitation. Female are at risk for developing depression (OR=1.41-1.61; CI=1.51), yet they are likely to report for better health status (OR=1.54; CI=1.45-1.64) than males. Asian (OR=0.56; CI=0.39-0381), Black or African American (OR=0.54; CI=0.48-0.60) are less likely to be diagnosed with depressive disorder than White. Discussion/Recommendation: Physical and cognitive changes that come with aging can pose challenge, as functional capacity diminishes in home environment. Further research should be explored in longitudinal studies on mobility and improving quality of living among isolated aging population.

